# Effects of an Immersive 180° Video–Based Virtual Reality Instructional Module on Learning Satisfaction, Technology Acceptance, Motivation, and Quiz-Based Learning Achievement in Undergraduate Physical Therapy Students: Randomized Controlled Trial

**DOI:** 10.2196/94122

**Published:** 2026-08-14

**Authors:** Hyoshil Yoon, Yongwoo Lee

**Affiliations:** 1 Department of Physical Therapy, Graduate School, Sahmyook University, Seoul, Republic of Korea; 2 Department of Physical Therapy, Sahmyook University, 815 Hwarang-ro, Nowon-gu, Seoul, 01795, Republic of Korea, 82 01024469705

**Keywords:** virtual reality, physical therapy, physiotherapy, learning satisfaction, technology acceptance, learning motivation, undergraduate students

## Abstract

**Background:**

Virtual reality (VR) offers immersive learning opportunities in health professions education, yet its effects on learner experience and short-term learning outcomes in education on execution of musculoskeletal special tests remain underexplored.

**Objective:**

This randomized controlled trial evaluated the effects of an immersive 180° video–based VR instruction, compared with conventional 2D video–based instruction, on undergraduate physical therapy students’ learning satisfaction, technology acceptance, learning motivation, and quiz-based learning achievement, reflecting short-term procedural knowledge.

**Methods:**

Undergraduate physical therapy students were randomly assigned to either the immersive 180° video–based VR instruction group (VR group) or the conventional 2D video–based instruction group (control group). Both groups received identical instructional content on 4 musculoskeletal special tests (pronator teres, Hawkins-Kennedy, Yergason, and Neer tests). The VR group participated in an immersive 180° video–based instructional module delivered via a head-mounted display, whereas the control group viewed the same content on a standard monitor. Participants completed 4 instructional sessions within a single day, each lasting approximately 3 to 4 minutes. The primary outcome was learning satisfaction. Secondary outcomes were technology acceptance, learning motivation, and quiz-based learning achievement. These outcomes were assessed before and after the intervention using validated questionnaires and a 12-item quiz. Between-group posttest differences were primarily analyzed using analysis of covariance (ANCOVA) with pretest scores as covariates. Supplementary within-group and unadjusted between-group analyses were also performed.

**Results:**

Fifty-two students were randomly assigned to receive either immersive 180° video–based VR instruction (VR group; n=28) or conventional 2D video–based instruction (control group; n=24) on 4 musculoskeletal special tests, with all 4 instructional sessions completed within a single day. After adjustment for baseline scores, the VR group showed significantly higher learning satisfaction (adjusted mean 107.04 vs 93.28; *F*_1,49_=13.710; *P*=.001; ηp^2^=0.219) and technology acceptance (adjusted mean 74.44 vs 68.03; *F*_1,49_=4.402; *P*=.04; ηp^2^=0.082) than the control group. Adjusted between-group differences were not significant for quiz-based learning achievement (*F*_1,49_=1.175; *P*=.28). For learning motivation, the homogeneity of regression slopes assumption was violated; therefore, the adjusted estimate was interpreted descriptively and did not indicate a clear between-group advantage (*F*_1,48_=1.729; *P*=.20).

**Conclusions:**

Compared with conventional 2D video–based instruction, immersive 180° video–based VR instruction showed a clear advantage in learning satisfaction, while technology acceptance also favored the VR group in exploratory secondary analysis, among undergraduate physical therapy students. Findings for learning motivation and quiz-based learning achievement were less conclusive. These results support immersive video as a supplementary educational approach rather than as evidence of superior psychomotor skills training.

## Introduction

With the rapid advancement of digital technologies, technology-enhanced learning has emerged as a transformative approach in health professions education [1]. In particular, virtual reality (VR) has gained recognition as a useful educational tool, creating immersive and learner-centered learning experiences for both instructors and students [2-4].

The use of immersive technologies in health professions education has expanded substantially, with applications ranging from procedural simulation to skills training. Recent developments in head-mounted displays have increased accessibility and instructional flexibility in these learning environments [4-7].

Several studies have found that VR-enhanced learning environments increase learner enjoyment and engagement, particularly in anatomy education [7]. According to self-determination theory, such enjoyable and autonomous learning contexts can foster intrinsic motivation and improve educational outcomes [8].

In physical therapy education, where hands-on clinical skills are essential, immersive digital media may support early familiarization with procedural steps by allowing learners to observe anatomical landmarks, examiner hand placement, patient positioning, and movement sequence in a more engaging format [9-12]. In particular, musculoskeletal special tests—used to diagnose orthopedic conditions—are fundamental skills for physical therapists in clinical settings [13]. Given the complex anatomical relationships involved in these assessments, learners must be able to understand when and how these tests are performed with both accuracy and confidence. Emerging evidence suggests that immersive video–based and other VR-supported educational approaches may be useful in physiotherapy education, although their effects may vary according to the level of interactivity and instructional design [10,14]. Rather than serving as a comprehensive practical skills curriculum, immersive 180° video–based VR may be particularly useful as a brief supplementary instructional approach for early familiarization with clinical procedures. By providing a first-person view of anatomical landmarks, examiner hand placement, patient positioning, and procedural sequence, such immersive video may support concrete observational exposure and learner engagement, which are broadly consistent with experiential learning principles [15]. In addition, a standardized immersive format may help novice learners focus on key procedural elements before hands-on practice [16,17]. From a contemporary instructional design perspective, immersive 360° or spherical video should be distinguished from fully interactive VR simulation, as immersion alone does not provide the same level of interactivity, feedback, or practice opportunity [18]. Within this fidelity spectrum, immersive 180° video–based VR can be positioned between conventional 2D video and fully interactive VR simulation, offering greater visual immersion than standard video but without task manipulation, haptic feedback, or real-time performance feedback. Thus, the present intervention was conceptualized as an immersive video–based instructional module rather than as fully interactive VR simulation and was designed to support early familiarization with musculoskeletal special tests rather than to provide comprehensive psychomotor skills training.

Although numerous VR studies have explored its application in rehabilitation for patients with stroke or degenerative arthritis [19,20], evidence regarding immersive 180° video–based VR instruction for undergraduate physical therapy students remains limited [10,16]. The pedagogical potential of immersive 180° video–based VR delivered through a head-mounted display, as distinct from fully interactive VR simulation, remains underexplored in musculoskeletal special tests education. Further evidence is needed to support its curricular integration.

Therefore, this study aimed to compare the effects of an immersive 180° video–based VR instructional module vs conventional 2D video–based instruction on learning satisfaction, technology acceptance, learning motivation, and quiz-based learning achievement, reflecting short-term procedural knowledge, among undergraduate physical therapy students receiving education on musculoskeletal special tests.

## Methods

### Study Design

This study adopted a single-blinded, parallel-group randomized controlled trial design to compare the effects of an immersive 180° video–based VR instructional module versus conventional 2D video–based instruction on key learning outcomes in undergraduate physical therapy students. No important changes to the trial design, eligibility criteria, intervention procedures, or outcome assessment procedures were made after trial commencement. The study protocol was registered at ClinicalTrials.gov (NCT07003217) on June 3, 2025, after participant recruitment and data collection had been completed; therefore, the trial registration was retrospective. The delayed registration occurred because the study was initially conducted as a single-institution educational intervention study and was not prospectively registered before recruitment; public trial registration was completed later during manuscript preparation. ClinicalTrials.gov is a primary registry of the World Health Organization International Clinical Trials Registry Platform and is recognized by the International Committee of Medical Journal Editors. The study was reported in accordance with the CONSORT-EHEALTH (Consolidated Standards of Reporting Trials of Electronic and Mobile Health Applications and Online Telehealth) V1.6 checklist [21], and the completed checklist is provided as Checklist 1.

### Ethical Considerations

This study was conducted in accordance with the ethical principles of the Declaration of Helsinki. Ethical approval was obtained from the institutional review board of Sahmyook University (SYU 2023-11-001-002). All participants were informed about the purpose and procedures of the study and provided written informed consent prior to participation.

### Participants

A total of 53 undergraduate physical therapy students were recruited. Participants were recruited from third- and fourth-year undergraduate physical therapy students because these students had already received theoretical instruction on the target musculoskeletal special tests. Inclusion criteria were (1) enrollment in the physical therapy program, (2) prior theoretical instruction on the 4 target musculoskeletal special tests, and (3) no “severe” symptoms on the Simulator Sickness Questionnaire (SSQ; Multimedia Appendix 1). Exclusion criteria included neurological or musculoskeletal conditions limiting VR use, pregnancy, or refusal to participate.

The required sample size was estimated using G*Power (version 3.1.9.7; Heinrich Heine University). Because prior effect size estimates specific to immersive 180° video–based VR instruction in undergraduate physical therapy education were limited, a moderate-to-large effect size (Cohen *d*=0.70) was selected for the sample size calculation. Using a 2-tailed test with a statistical power of 0.80 and a significance level of .05, the analysis indicated that a minimum of 52 participants was required.

### Randomization and Blinding

Participants were randomly assigned to either the immersive 180° video–based VR instruction group (VR group) or the conventional 2D video–based instruction group (control group) using a computer-generated randomization sequence. Allocation was performed by an independent researcher not involved in data collection. Outcome assessors were blinded to group allocation.

### Educational Content: Musculoskeletal Special Tests

The intervention targeted 4 clinically relevant upper extremity special tests commonly used in orthopedic physical therapy. These were selected based on their diagnostic utility and inclusion in standard clinical curricula.

- Pronator teres test: used to assess compression of the median nerve by the pronator teres muscle, distinguishing pronator teres syndrome from carpal tunnel syndrome.
- Hawkins-Kennedy test: a provocative test for subacromial impingement, conducted through passive internal rotation of the shoulder at 90° of flexion.
- Yergason test: evaluates the integrity of the transverse humeral ligament and identifies pathology involving the long head of the biceps tendon or the glenoid labrum.
- Neer test: detects impingement involving the supraspinatus or long head of the biceps brachii through passive forward flexion of the internally or externally rotated arm.

Educational modules emphasized anatomical landmarks, examiner hand placement, patient positioning, proper test execution, and interpretation of positive signs. The 4 musculoskeletal special tests were established clinical examination procedures; however, the instructional video content used was custom-produced for the study. The procedures were recorded based on standard musculoskeletal examination methods and then produced as immersive 180° VR video content by a professional video production company. The instructional videos were not commercially available educational materials. Instructional content was standardized across both groups to ensure consistency. This intervention was designed as a brief supplementary instructional module rather than a comprehensive practical skills curriculum. Its educational purpose was to provide standardized immersive observational exposure to key procedural elements of each special test, including anatomical landmarks, examiner hand placement, patient positioning, movement sequence, and interpretation of positive signs. Repeated hands-on practice, performance feedback, and formal retention assessment were not incorporated into the intervention design.

### Intervention Procedures

Participants completed 4 special test instructional sessions within a single day. To accommodate all participants, data collection was conducted over 4 consecutive days. Each session included 1 test and lasted approximately 3 to 4 minutes. The single-day format was selected to align with the study aim of evaluating immediate learner experience and short-term procedural knowledge after a brief supplementary familiarization module, while minimizing variability in exposure conditions across participants. Interventions were conducted in a laboratory setting.

#### Immersive 180° Video–Based VR Instruction (VR Group)

Participants wore a head-mounted display (Pico 4 Enterprise [Pico Technology]) and used handheld controllers to access prerecorded immersive 180° VR videos for each special test. All participants were instructed on device use, hygiene, and safety protocols prior to the sessions. The immersive video content was custom-produced for this study by a professional video production company and was preloaded onto the head-mounted displays before each session. Within the headset environment, participants used the handheld controllers to adjust the viewing direction (up, down, left, and right), zoom in and out, pause and replay the videos, and move to the next segment. Thus, the intervention allowed limited user control over viewpoint and playback, but it did not include virtual object manipulation, hand tracking, haptic feedback, task-performance tracking, or real-time performance feedback. Detailed production specifications, including camera model, recording resolution, frame rate, stabilization software, and audio-production characteristics, were not available to the authors because the videos were externally produced by a professional vendor. Device-level playback characteristics such as latency and rendering-pipeline parameters were not independently measured in this study.

#### Conventional 2D Video–Based Instruction (Control Group)

Participants received the same instructional content via 2D video on a standard monitor while seated in a classroom setting. Session duration and frequency were identical to those of the VR group.

Accordingly, the 4 short sessions were intended to support early procedural familiarization in a standardized format rather than to replace hands-on skills practice. A summary of the intervention is shown in Table 1.

**Table 1.** Purpose and procedures of musculoskeletal special tests included in VR^a^ group and control group.

Special test	Clinical purpose	Testing procedure description
Pronator teres test	To detect median nerve compression by the pronator teres muscle (pronator teres syndrome)	With the elbow flexed to 90°, the patient performs forearm pronation while the examiner applies counter-resistance.
Hawkins-Kennedy test	To identify subacromial impingement syndrome	With the shoulder and elbow flexed to 90°, the examiner passively internally rotates the shoulder.
Yergason test	To assess stability of the biceps tendon and integrity of the glenoid labrum	With the elbow flexed to 90° and adducted, the patient attempts to supinate and externally rotate the forearm against resistance.
Neer test	To detect impingement of the supraspinatus or long head of the biceps brachii	The examiner passively flexes the shoulder with the arm internally or externally rotated, noting pain provocation.

a VR: virtual reality.

### Outcome Measures

All outcomes were assessed before (pretest) and immediately after (posttest) the single-day intervention per participant using validated instruments, as described in Multimedia Appendices 2-5. All outcome assessments were conducted face-to-face in the laboratory or classroom setting and were not administered through an online platform. The primary outcome was learning satisfaction. Secondary outcomes were technology acceptance, learning motivation, and quiz-based learning achievement.

- Learning satisfaction: measured by a 19-item scale evaluating system experience, emotional response, and learning satisfaction (Cronbach α=0.91), using a 7-point Likert scale [1,22]
- Technology acceptance: assessed with 13 items (6 for usefulness, 7 for ease of use), validated in prior studies (Cronbach α=0.95 and 0.94), using a 7-point Likert scale [23]
- Learning motivation: evaluated through 7 items measuring interest, perceived importance, and intention to learn (Cronbach α=0.79), also on a 7-point Likert scale [23]
- Quiz-based learning achievement: assessed with a 12-item quiz (short-answer and multiple-choice), covering all 4 tests. Questions were reviewed for content validity by 2 physical therapy education experts. Identical quizzes were used for pre- and posttesting. This measure was intended to assess short-term procedural knowledge related to the 4 special tests rather than direct psychomotor performance. No delayed posttest or retention assessment was conducted

### Participant Feedback

Participant feedback was collected immediately after the intervention using open-ended written response items included in the postintervention questionnaire. The feedback was optional and was intended to provide descriptive contextual information regarding participants’ experiences with the instructional method. Participants were asked to comment on perceived advantages, disadvantages, discomfort, and overall impressions of the instructional format. Responses were anonymized before analysis. Two researchers independently reviewed the written responses, identified recurring meaning units, and grouped similar responses into descriptive themes. Any differences in theme grouping were resolved through discussion. Because the feedback was collected through brief written responses rather than interviews or focus groups, the findings were summarized descriptively and were not intended to represent a full qualitative study.

### Statistical Analysis

Data were analyzed using SPSS (version 22.0; IBM Corp), with statistical significance set at *P*<.05. Baseline homogeneity was assessed using independent *t* tests or Mann-Whitney *U* tests for continuous variables and *χ*^2^ tests for categorical variables, as appropriate. The primary between-group analysis was conducted using analysis of covariance (ANCOVA) on posttest scores, with the corresponding pretest scores entered as covariates, after verifying the assumptions of homogeneity of variances and homogeneity of regression slopes. Partial eta-squared (ηp^2^) was reported as an effect size for the ANCOVA results. Supplementary analyses included paired *t* tests (or Wilcoxon signed rank tests for nonnormal data) for within-group pre-post changes and independent *t* tests (or Mann-Whitney *U* tests for nonnormal data) for unadjusted between-group comparisons. Because learning satisfaction was specified as the primary outcome and the remaining outcomes were secondary, no formal multiplicity adjustment was applied. The secondary outcomes were considered exploratory and were interpreted more cautiously than the primary outcome.

## Results

### Participant Characteristics and Baseline Comparisons

**Figure 1.**
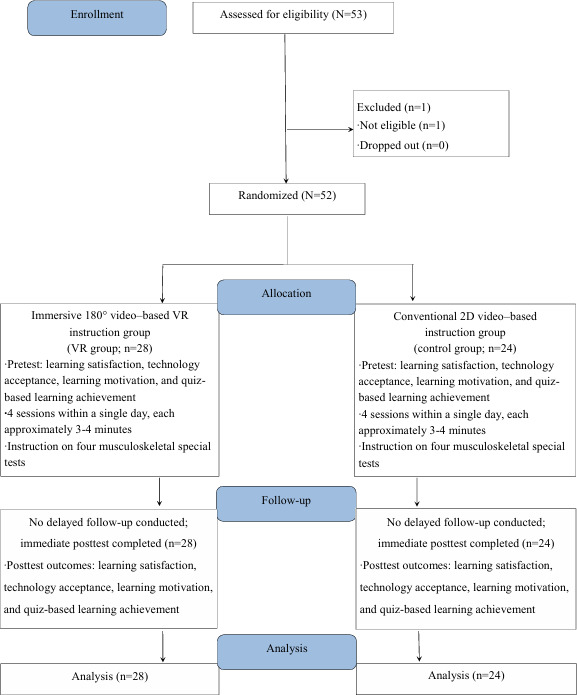
CONSORT (Consolidated Standards of Reporting Trials) participant flow diagram.

Of 53 students recruited, 1 was excluded because the eligibility criteria were not met, and 52 were randomly assigned to the VR group (n=28; 10 males and 18 females) or the control group (n=24; 13 males and 11 females). The CONSORT participant flow diagram is presented in Figure 1. All randomized participants received the assigned intervention and were included in the primary outcome analysis. Baseline demographic characteristics showed a significant between-group difference in age, but no significant differences in height, weight, SSQ scores, or sex distribution (age: *P*=.02; height: *P*=.32; weight: *P*=.94; SSQ total score: *P*=.61; sex: *P*=.18) (Table 2).

**Table 2.** Baseline characteristics of participants (N=52). Between-group comparisons for continuous variables were conducted using independent *t* tests.

Variable	Virtual reality group (n=28)	Control group (n=24)	*P* value
Age (years), mean (SD)	21.68 (1.59)	24.00 (4.91)	.02
Height (cm), mean (SD)	166.82 (8.25)	169.29 (8.79)	.32
Weight (kg), mean (SD)	62.07 (12.30)	62.33 (12.22)	.94
SSQ^a^ total score, mean (SD)	4.67 (6.77)	3.71 (5.60)	.61
Male:female (ratio)	10:18	13:11	.18^b^

a SSQ: Simulator Sickness Questionnaire.

b *P* value from *χ*2 test for sex distribution.

### Baseline Equivalence of Outcome Measures

Preintervention scores for learning satisfaction (*P*=.30), technology acceptance (*P*=.16), learning motivation (*P*=.68), and quiz-based learning achievement (*P*=.26) were statistically equivalent between the 2 groups, confirming baseline homogeneity (Table 3).

**Table 3.** Baseline equivalence of outcome measures between groups (N=52).

Outcome variable	Virtual reality group, mean (SD)	Control group, mean (SD)	*P* value
Learning satisfaction	89.96 (12.30)	83.54 (11.32)	.30
Technology acceptance	63.43 (11.84)	59.33 (8.22)	.16
Learning motivation^a^	40.29 (5.30)	40.72 (3.46)	.68
Quiz-based learning achievement	0.68 (0.98)	0.98 (0.91)	.26

a For learning motivation, the unequal variances *t* test result was used because Levene test indicated unequal variances.

### Learning Satisfaction

An ANCOVA on posttest scores, controlling for baseline, revealed a significant effect for group. The VR group (adjusted mean 107.04) scored significantly higher than the control group (adjusted mean 93.28; *F*_1,49_=13.710; *P*=.001; ηp^2^=0.219), with an adjusted mean difference of 13.762 (95% CI 6.293-21.231). An unadjusted *t* test on posttest scores confirmed this significant difference (*P*<.001) (Tables 4 and 5).

**Table 4.** Primary analysis: analysis of covariance (ANCOVA) results for posttest scores (N=52).

Variable and group	Adjusted mean^a^	Adjusted mean difference (95% CI)	Between-group ANCOVA, *F* test (*df*)	*P* value	Partial eta-squared (ηp^2^)
Learning satisfaction		13.762 (6.293 to 21.231)	13.710 (1, 49)	.001	0.219
Virtual reality	107.04				
Control	93.28				
Technology acceptance		6.410 (0.270 to 12.549)	4.402 (1, 49)	.04	0.082
Virtual reality	74.44				
Control	68.03				
Learning motivation		1.688 (−1.131 to 4.508)^b^	1.729 (1, 48)^b^	.20^b^	0.035
Virtual reality	43.66				
Control	41.98				
Quiz-based learning achievement		0.833 (−0.712 to 2.378)	1.175 (1, 49)	.28	0.023
Virtual reality	5.94				
Control	5.11				

a Adjusted mean: adjusted posttest mean.

b Homogeneity of regression slopes was violated for learning motivation (interaction: *F*_1,48_=4.328; *P*=.04; ηp2=0.083); therefore, this result should be interpreted with caution and was retained as a descriptive adjusted estimate rather than a confirmatory ANCOVA finding.

**Table 5.** Supplementary analysis: unadjusted within-group and between-group comparisons (N=52).

Variable and group	Pretest	Posttest	Within-group comparison^a^ (pre- vs posttest), *t* test (*df*)	Within-group comparison^a^ (pre- vs posttest), *P* value	Between-group comparison^b^ (posttest), *t* test (*df*)	Between-group comparison^b^ (posttest), *P* value
Learning satisfaction, mean (SD)					4.508 (50)	<.001
Virtual reality	89.96 (12.30)	107.82 (14.19)	−6.398 (27)	<.001		
Control	83.54 (11.32)	91.25 (11.97)	−3.286 (23)	.003		
Technology acceptance, mean (SD)					2.828 (50)	.007
Virtual reality	63.43 (11.84)	75.29 (10.86)	−4.053 (27)	<.001		
Control	59.33 (8.22)	66.75 (10.84)	−3.798 (23)	.001		
Learning motivation, mean (SD)					1.721 (50)	.09
Virtual reality	40.29 (5.30)	44.07 (5.02)	−2.444 (27)	.02		
Control	40.72 (3.46)	41.71 (4.83)	−1.399 (23)	.18		
Quiz-based learning achievement^c^, median (IQR)					267.500	.21^d^
Virtual reality	0.00 (0.00‐2.00)	5.75 (3.63‐7.88)	−4.625^e^	<.001		
Control	1.00 (0.00‐2.00)	5.00 (2.63-8.00)	−3.682^e^	<.001		

a Within-group comparison: paired *t* test or Wilcoxon signed rank test.

b Between-group comparison: independent *t* test.

c Nonparametric tests were used for quiz-based learning achievement due to nonnormality.

d Between-group comparison: Mann-Whitney *U* test.

e *z *value from Wilcoxon signed rank test.

### Technology Acceptance

After adjusting for pretest scores, the VR group (adjusted mean 74.44) showed significantly higher acceptance than the control group (adjusted mean 68.03; *F*_1,49_=4.402; *P*=.04; ηp^2^=0.082) with an adjusted mean difference of 6.41 (95% CI 0.270-12.549). This finding was consistent with the unadjusted *t* test on posttest scores (*P*=.007) (Tables 4 and 5).

### Learning Motivation

The ANCOVA assumption of homogeneity of regression slopes was violated (interaction effect: *F*_1,48_=4.328; *P*=.04). At the mean baseline, the adjusted difference between the VR group (adjusted mean 43.66) and the control group (adjusted mean 41.98) was not significant *(F*_1,48_=1.729; *P*=.20; ηp^2^=0.035) with an adjusted mean difference of 1.688 (95% CI −1.131 to 4.508), a finding consistent with the unadjusted *t* test result (*P*=.09) (Tables 4 and 5).

### Quiz-Based Learning Achievement

There was no significant difference in quiz-based learning achievement between the VR group (adjusted mean 5.94) and the control group (adjusted mean 5.11) after controlling for pretest scores (*F*_1,49_=1.175; *P*=.28; ηp^2^=0.023) with an adjusted mean difference of 0.833 (95% CI **−**0.712 to 2.378). An unadjusted Mann-Whitney *U* test on posttest scores was also not statistically significant (*P*=.21) (Tables 4 and 5).

### Participant Feedback on Instructional Methods

Written feedback was collected from 22 of 28 (79%) participants in the VR group and 10 of 24 (42%) in the control group. VR group responses emphasized increased engagement, realism, and learning interest. However, several participants noted visual discomfort or fatigue after prolonged use of the head-mounted display. Control group participants found the 2D video format familiar and easy to follow but reported perceived limitations in motivation and interactivity. A descriptive summary of participant responses is provided in Table 6.

**Table 6.** Participants’ feedback on instructional methods (n=52).

Group	Respondents, n (%)	Themes identified
Virtual reality group (n=28)	22 (79)	Enhanced realism, 3D learning, personalized viewpoints, close observation, positive engagement. Some discomfort (eg, dizziness, visual fatigue, headset weight)
Control group (n=24)	10 (42)	Familiar video format, easy to follow. Reported limitations: lack of interactivity, shared viewing, perceived monotony

## Discussion

This study compared a low-interactivity immersive 180° video–based VR instruction with conventional 2D video instruction for musculoskeletal special test education in undergraduate physical therapy students. The immersive video module was associated with higher learning satisfaction than 2D video, and technology acceptance also favored the VR group in exploratory secondary analysis, whereas between-group differences were not significant for learning motivation or quiz-based learning achievement.

First, the enhanced learning satisfaction observed in the VR group highlights the pedagogical value of immersive viewing environments, which may help learners more intuitively understand anatomical landmarks, examiner hand placement, patient positioning, and procedural sequence. These findings align with principles of experiential learning theory, which emphasize the role of concrete experience and reflective observation in knowledge construction [15]. This interpretation is also broadly consistent with previous studies suggesting that immersive learning environments can increase learner engagement and perceived educational value [6,16].

Second, the higher technology acceptance observed in the VR group may reflect the immersive first-person viewing experience, focused attention, and perceived novelty afforded by the head-mounted display environment rather than fully interactive simulation features [5,6]. From a self-determination theory perspective, immersive and autonomy-supportive learning environments may enhance learner interest and engagement [8]. However, because the present intervention consisted of prerecorded 180° videos with limited interactivity, these findings should not be interpreted as evidence for the effects of fully interactive VR simulation.

Third, although the VR group showed a significant within-group increase in learning motivation, the between-group difference was not statistically significant. Similarly, both groups showed within-group increases in quiz-based learning achievement, but the adjusted between-group difference was not statistically significant. Importantly, the measured outcomes did not include direct assessment of clinical skill performance, such as Objective Structured Clinical Examination–based evaluation, checklist-based observation, or other performance-based assessments. Accordingly, the present findings more strongly support the value of immersive 180° video–based VR for learner experience outcomes than for superior short-term quiz performance. Because quiz-based learning achievement was assessed using an identical pre- and posttest administered within a single day, the observed gains should be interpreted cautiously, as they may partly reflect test-retest or recall effects rather than differences attributable solely to instructional modality. In addition, because multiple outcomes were examined and adjusted between-group differences were not significant for all secondary outcomes, findings for learning motivation and quiz-based learning achievement should be interpreted cautiously.

Despite these strengths, several limitations should be considered. First, the present VR intervention used prerecorded immersive 180° videos with limited interactivity and no haptic feedback. Therefore, it was conceptually distinct from fully interactive simulation-based VR and was limited in its ability to support psychomotor skill development, task manipulation, or real-time performance feedback [10,24]. Detailed production specifications for the externally produced immersive videos were not available to the authors; therefore, some technical parameters of content capture and postproduction could not be fully reported. Future studies should investigate whether more interactive systems, including hand tracking, gesture-based interaction, or haptic feedback, produce different educational effects [24,25].

Second, the intervention was brief, with 4 instructional sessions completed within a single day. As a result, the study did not examine repeated practice, skill transfer, or longer-term retention. Future research should incorporate longitudinal designs, repeated exposure, and performance-based assessments to better evaluate the role of immersive technologies in musculoskeletal special test education [10,16].

Third, the primary and several secondary outcomes were based on self-reported questionnaires. Although outcome assessors were blinded to group allocation, participant blinding was not feasible because learners could clearly recognize whether they were using a head-mounted display or viewing a conventional 2D video. Therefore, the higher learning satisfaction and technology acceptance observed in the VR group may have been influenced by novelty effects, expectancy effects, or differences in perceived technological sophistication. Future studies should include additional objective or performance-based outcomes and, where possible, comparator interventions that better control for novelty and attention.

Fourth, this study did not control for potential differences in baseline academic ability, spatial cognition, or prior clinical exposure, which may have influenced the observed outcomes. Future research should consider these learner characteristics and explore whether specific subgroups benefit more from immersive video–based instruction [24].

Fifth, this was a single-institution study conducted with undergraduate physical therapy students in Korea, and a significant baseline age difference was observed between groups. Although the primary ANCOVA adjusted for baseline outcome scores, age was not included as a covariate; therefore, residual confounding related to age cannot be fully excluded. Therefore, the findings should be generalized cautiously to other countries, institutions, or educational systems, as well as to programs with different curricula or different levels of prior VR exposure. Future multicenter studies across diverse educational contexts are needed to examine the broader applicability of immersive 180° video–based VR instruction.

Nevertheless, this study provides preliminary evidence that an immersive 180° video–based VR format may serve as a useful supplementary educational tool in physical therapy education [10,16]. In the present study, the most robust advantage was observed for learning satisfaction, while technology acceptance also favored the VR group in exploratory secondary analysis. This type of immersive video–based instruction may be particularly useful in situations where opportunities for direct observation are limited by resource constraints or accessibility barriers, while still being conceptually distinct from fully interactive simulation-based VR [10].

To maximize the instructional potential of immersive technologies in health professions education, future studies should also examine extended reality and spatial computing applications that integrate immersive visualization with real-time interaction, spatial mapping, and task-specific feedback, as well as repeated practice and performance-based assessments.

This randomized controlled trial demonstrated that immersive 180° video–based VR instruction may serve as a useful supplementary educational approach in undergraduate physical therapy education. Compared to conventional 2D video instruction, immersive 180° video–based VR instruction showed a clear advantage in learning satisfaction, while an exploratory advantage was observed for the VR group in technology acceptance. Findings for learning motivation and quiz-based learning achievement were less conclusive.

These findings contribute to the growing body of evidence supporting the pedagogical utility of immersive digital tools in health professions education. In the present study, the findings are most appropriately interpreted as support for learner experience outcomes rather than as evidence of superior psychomotor skills training. In this study, the immersive 180° video–based VR environment provided a safe and engaging context for students to observe procedural content related to musculoskeletal special tests; however, it did not include interactive simulation features such as hand tracking, haptic feedback, task-performance tracking, or real-time feedback.

Because this study used pre-recorded 180° VR content without tactile or interactive functionality, future research should examine whether more advanced VR systems, including haptic feedback, gesture recognition, and AI-guided instruction, produce additional benefits for psychomotor performance, skill transfer, or retention. However, because the intervention was brief and low in interactivity, and quiz-based learning achievement was assessed using a short-term quiz rather than direct performance-based assessment, the present findings should not be interpreted as evidence of improved psychomotor learning or skill transferability to clinical practice.

As physical therapy education continues to embrace digital transformation, immersive 180° video–based VR instruction may be integrated as a supplementary modality to reinforce procedural knowledge within similar undergraduate physical therapy education contexts. However, because this study was conducted at a single institution in Korea, the findings should be generalized cautiously to other countries or educational systems. Future research should explore the long-term effects of immersive 180° video–based VR instruction, using spaced practice, Objective Structured Clinical Examination–based or other performance-based assessments, more interactive VR modalities, and longer-term retention measures.

## Supplementary material

10.2196/94122 Multimedia Appendix 1 Simulator Sickness Questionnaire (SSQ) items and severity scores.

10.2196/94122 Multimedia Appendix 2 Learning satisfaction questionnaire.

10.2196/94122 Multimedia Appendix 3 Technology Acceptance Questionnaire.

10.2196/94122 Multimedia Appendix 4 Learning motivation questionnaire.

10.2196/94122 Multimedia Appendix 5 Quiz-based learning achievement test.

10.2196/94122 Checklist 1 CONSORT-EHEALTH Checklist.

## Abbreviations

ANCOVA: analysis of covariance
CONSORT-EHEALTH: Consolidated Standards of Reporting Trials of Electronic and Mobile Health Applications and Online Telehealth
SSQ: Simulator Sickness Questionnaire
VR: virtual reality
