# Supplementary material for: Effects of an Immersive 180° Video–Based Virtual Reality Instructional Module on Learning Satisfaction, Technology Acceptance, Motivation, and Quiz-Based Learning Achievement in Undergraduate Physical Therapy Students: Randomized Controlled Trial
Source: JMIR Med Educ. 2026 Aug 14;12:e94122. doi: 10.2196/94122 (PMC13476000; doi:10.2196/94122)
Supplement: Supplementary file 2 [file mededu-v12-e94122-s001.docx]

**Multimedia Appendix 1. Simulator Sickness Questionnaire (SSQ) Items and Severity Scores**

| Symptom | Severity Score (0–3) |
| --- | --- |
| General discomfort | 0–3 |
| Fatigue | 0–3 |
| Headache | 0–3 |
| Eyestrain | 0–3 |
| Difficulty focusing the eyes | 0–3 |
| Increased salivation | 0–3 |
| Sweating | 0–3 |
| Nausea | 0–3 |
| Difficulty concentrating | 0–3 |
| Fullness of head | 0–3 |
| Blurred vision | 0–3 |
| Dizziness (eyes open) | 0–3 |
| Dizziness (eyes closed) | 0–3 |
| Vertigo | 0–3 |
| Stomach awareness | 0–3 |
| Burping | 0–3 |
| Total score | Range: 0–48 |

Note: SSQ = Simulator Sickness Questionnaire; Severity: 0 = none, 1 = slight, 2 = moderate, 3 = severe.
