# Supplementary material for: Effects of an Immersive 180° Video–Based Virtual Reality Instructional Module on Learning Satisfaction, Technology Acceptance, Motivation, and Quiz-Based Learning Achievement in Undergraduate Physical Therapy Students: Randomized Controlled Trial
Source: JMIR Med Educ. 2026 Aug 14;12:e94122. doi: 10.2196/94122 (PMC13476000; doi:10.2196/94122)
Supplement: Supplementary file 3 [file mededu-v12-e94122-s002.docx]

**Multimedia Appendix 2. Learning satisfaction questionnaire**

2 -1. Learning satisfaction questionnaire

**Please read the following questions carefully and mark (∨) the answer that best matches your opinion.**

(1 = not at all, 2 = no, 3 = somewhat not, 4 = moderate, 5 = somewhat, 6 = yes, 7 = very much).

| **Learning satisfaction questionnaire items** | **1** | **2** | **3** | **4** | **5** | **6** | **7** |
| --- | --- | --- | --- | --- | --- | --- | --- |
| **scale 1: Experience using previous learning systems** | | | | | | | |
| 1) The previous learning system was easy to use. |  |  |  |  |  |  |  |
| 2) After participating in the previous training, I was able to fully learn how to use the learning system in a short period of time. |  |  |  |  |  |  |  |
| 3) Previous learning systems always help in proper understanding of the class topics. |  |  |  |  |  |  |  |
| **scale 2: Feelings about previous learning systems** | | | | | | | |
| 4) When I was guided through my previous studies, I liked learning through the previous learning system. |  |  |  |  |  |  |  |
| 5) I would like to know if I can apply the previous learning system to study other subjects to improve my learning performance. |  |  |  |  |  |  |  |
| 6) I want to learn through the education of the previous learning system not only today but also in the future. |  |  |  |  |  |  |  |
| 7) I would recommend the previous course to others. |  |  |  |  |  |  |  |
| 8) The use of previous learning instruction makes this learning activity more interesting. |  |  |  |  |  |  |  |
| 9) The learning system guidance provided in the previous lessons was easy to understand and follow. |  |  |  |  |  |  |  |
| 10) I tried to follow the learning guide given while the learning was in progress. |  |  |  |  |  |  |  |
| **scale 3: satisfaction with learning approach** | | | | | | | |
| 11) Previous learning activities will help me better understand how to define and classify learning topics. |  |  |  |  |  |  |  |
| 12) I tried to observe the learning topic better while I was learning. |  |  |  |  |  |  |  |
| 13) Although it was not easy to complete all the previous learning activities, it was still easy to understand the learning topic. |  |  |  |  |  |  |  |
| 14) Education through previous learning is more challenging and more interesting than traditional teaching methods. |  |  |  |  |  |  |  |
| 15) I was able to learn new knowledge through previous studies. |  |  |  |  |  |  |  |
| 16) I had a hard time using the previous learning system because it was new and different from the existing learning methods. |  |  |  |  |  |  |  |
| 17) The guidance provided for the previous learning system helped in defining and learning the characteristics of the learning topic. |  |  |  |  |  |  |  |
| 18) The guidance provided for the previous learning system was helpful in observing the content of the learning topic. |  |  |  |  |  |  |  |
| 19) When I used my previous learning system, I learned how to approach and implement learning. |  |  |  |  |  |  |  |

2 -2. Learning satisfaction questionnaire

**Please read the following questions carefully and mark (∨) the answer that best matches your opinion.**

(1 = not at all, 2 = no, 3 = somewhat not, 4 = moderate, 5 = somewhat, 6 = yes, 7 = very much).

| **Learning satisfaction questionnaire items** | **1** | **2** | **3** | **4** | **5** | **6** | **7** |
| --- | --- | --- | --- | --- | --- | --- | --- |
| **Scale 1: Experience using VR (virtual reality)-based learning systems** | | | | | | | |
| 1) This VR learning system was easy to use. |  |  |  |  |  |  |  |
| 2) After participating in this VR learning, I was able to fully learn how to use the learning system in a short period of time. |  |  |  |  |  |  |  |
| 3) This VR learning system always helps in properly understanding the class topic. |  |  |  |  |  |  |  |
| **scale 2: Feelings about VR-based learning systems** | | | | | | | |
| 4) When I was guided through VR learning, I liked the fact that I was learning using a VR-based teaching method. |  |  |  |  |  |  |  |
| 5) I would like to know if I can apply this VR learning system to learning other subjects to improve my learning performance. |  |  |  |  |  |  |  |
| 6) I want to learn through VR education not only today but also in the future. |  |  |  |  |  |  |  |
| 7) I would recommend this course to others. |  |  |  |  |  |  |  |
| 8) The use of VR training makes this learning activity more interesting. |  |  |  |  |  |  |  |
| 9) The VR learning system guidance provided in this study was easy to understand and follow. |  |  |  |  |  |  |  |
| 10) I tried to follow the learning guide given while the learning was in progress. |  |  |  |  |  |  |  |
| **scale 3: satisfaction with learning approach** | | | | | | | |
| 11) This learning activity will help me better understand defining and classifying learning topics. |  |  |  |  |  |  |  |
| 12) I tried to observe the learning topic better while I was learning. |  |  |  |  |  |  |  |
| 13) Although it was not easy to complete all of these learning activities, it was still easy to understand the learning topic. |  |  |  |  |  |  |  |
| 14) Education through VR learning is more challenging and interesting than traditional educational methods. |  |  |  |  |  |  |  |
| 15) I was able to learn new knowledge through today’s lesson. |  |  |  |  |  |  |  |
| 16) I found it a bit difficult to use this VR learning system because it was new and different from the existing learning methods. |  |  |  |  |  |  |  |
| 17) The guidance provided for this VR learning helped define and learn the characteristics of the learning topic. |  |  |  |  |  |  |  |
| 18) The guidance provided for this VR learning was helpful in observing the content of the learning topic. |  |  |  |  |  |  |  |
| 19) When I used this VR learning system, I learned how to approach and implement learning. |  |  |  |  |  |  |  |
