# Supplementary material for: Effects of an Immersive 180° Video–Based Virtual Reality Instructional Module on Learning Satisfaction, Technology Acceptance, Motivation, and Quiz-Based Learning Achievement in Undergraduate Physical Therapy Students: Randomized Controlled Trial
Source: JMIR Med Educ. 2026 Aug 14;12:e94122. doi: 10.2196/94122 (PMC13476000; doi:10.2196/94122)
Supplement: Supplementary file 4 [file mededu-v12-e94122-s003.docx]

**Multimedia Appendix 3. Technology Acceptance Questionnaire**

3 -1. Technology Acceptance Questionnaire

**Please read the following questions carefully and mark (∨) the answer that best matches your opinion.**

(1 = not at all, 2 = no, 3 = somewhat not, 4 = moderate, 5 = somewhat, 6 = yes, 7 = very much).

| **Technology adoption** | **1** | **2** | **3** | **4** | **5** | **6** | **7** |  |
| --- | --- | --- | --- | --- | --- | --- | --- | --- |
| **Usefulness** | | | | | | | | |
| 1) Previous learning approaches enrich learning activities. |  |  |  |  |  |  |  |  |
| 2) Previous learning systems help in acquiring new knowledge. |  |  |  |  |  |  |  |  |
| 3) The learning mechanism provided through the learning system facilitated the learning process. |  |  |  |  |  |  |  |  |
| 4) Previous learning systems help me get useful information about what I need. |  |  |  |  |  |  |  |  |
| 5) Previous learning approaches help me understand the learning topic more clearly. |  |  |  |  |  |  |  |  |
| 6) Previous learning approaches are more useful than traditional learning methods. |  |  |  |  |  |  |  |  |
| **Ease of use** | | | | | | | | |
| 1) Learning about the previous learning system was not difficult. |  |  |  |  |  |  |  |  |
| 2) I was able to perfectly learn how to use the previous learning system in a short period of time. |  |  |  |  |  |  |  |  |
| 3) The learning activities conducted through the previous learning system were easy to understand and follow. |  |  |  |  |  |  |  |  |
| 4) I learned the previous learning system quickly. |  |  |  |  |  |  |  |  |
| 5) It was not difficult to participate in learning activities using the previous learning system. |  |  |  |  |  |  |  |  |
| 6) I found the previous learning system easy to use. |  |  |  |  |  |  |  |  |
| 7) In conclusion, the learning system used for the previous learning activities was easy to learn and use. |  |  |  |  |  |  |  |  |

3 -2. Technology Acceptance Questionnaire

**Please read the following questions carefully and mark (∨) the answer that best matches your opinion.**

(1 = not at all, 2 = no, 3 = somewhat not, 4 = moderate, 5 = somewhat, 6 = yes, 7 = very much).

| **Technology adoption** | **1** | **2** | **3** | **4** | **5** | **6** | **7** |
| --- | --- | --- | --- | --- | --- | --- | --- |
| **Usefulness** | | | | | | | |
| 1) This learning approach enriches learning activities. |  |  |  |  |  |  |  |
| 2) This learning system helps me gain new knowledge. |  |  |  |  |  |  |  |
| 3) The learning mechanism provided through the learning system facilitated the learning process. |  |  |  |  |  |  |  |
| 4) This learning system helps me get useful information on what I need. |  |  |  |  |  |  |  |
| 5) This learning approach helps me understand the learning topic more clearly. |  |  |  |  |  |  |  |
| 6) This learning approach is more useful than typical traditional learning methods. |  |  |  |  |  |  |  |
| **Ease of use** | | | | | | | |
| 1) Learning about this learning system was not difficult. |  |  |  |  |  |  |  |
| 2) I was able to perfectly learn how to utilize this learning system in a short period of time. |  |  |  |  |  |  |  |
| 3) The learning activities conducted through this learning system were easy to understand and follow. |  |  |  |  |  |  |  |
| 4) I learned this learning system quickly. |  |  |  |  |  |  |  |
| 5) It was not difficult to participate in learning activities using this learning system. |  |  |  |  |  |  |  |
| 6) I found this learning system easy to access. |  |  |  |  |  |  |  |
| 7) In conclusion, the learning system used for this learning activity was easy to learn and use. |  |  |  |  |  |  |  |
