# Supplementary material for: Effects of an Immersive 180° Video–Based Virtual Reality Instructional Module on Learning Satisfaction, Technology Acceptance, Motivation, and Quiz-Based Learning Achievement in Undergraduate Physical Therapy Students: Randomized Controlled Trial
Source: JMIR Med Educ. 2026 Aug 14;12:e94122. doi: 10.2196/94122 (PMC13476000; doi:10.2196/94122)
Supplement: Supplementary file 5 [file mededu-v12-e94122-s004.docx]

**Multimedia Appendix 4. Learning motivation questionnaire**

**Please read the following questions carefully and mark (∨) the answer that best matches your opinion.**

(1 = not at all, 2 = no, 3 = somewhat not, 4 = moderate, 5 = somewhat, 6 = yes, 7 = very much).

| **Motivation to learn** | **1** | **2** | **3** | **4** | **5** | **6** | **7** |
| --- | --- | --- | --- | --- | --- | --- | --- |
| 1) I find musculoskeletal special test interesting and valuable. |  |  |  |  |  |  |  |
| 2) I want to learn more about special test through this learning activity. |  |  |  |  |  |  |  |
| 3) It is absolutely necessary to learn about special tests. |  |  |  |  |  |  |  |
| 4) It is important to me to learn about special tests. |  |  |  |  |  |  |  |
| 5) Special tests are important to improve my abilities. |  |  |  |  |  |  |  |
| 6) I will actively seek out and learn information about special test. |  |  |  |  |  |  |  |
| 7) It is important for everyone to learn about special tests. |  |  |  |  |  |  |  |
