# Supplementary material for: Effects of an Immersive 180° Video–Based Virtual Reality Instructional Module on Learning Satisfaction, Technology Acceptance, Motivation, and Quiz-Based Learning Achievement in Undergraduate Physical Therapy Students: Randomized Controlled Trial
Source: JMIR Med Educ. 2026 Aug 14;12:e94122. doi: 10.2196/94122 (PMC13476000; doi:10.2196/94122)
Supplement: Supplementary file 6 [file mededu-v12-e94122-s005.docx]

**Multimedia Appendix 5. Quiz-based learning achievement test**

*** Read the following questions and write or choose the correct answer.**

| ***Special test*** | **Academic achievement assessment problem** |
| --- | --- |
| **Pronator teres test** | 1. What is the purpose of this test?  (____________________________________________________________)  2. What nerve passes under (between) the two heads of the pronator teres?  (_______________________________________________________________)  3. In what range of hands do signs such as numbness and tingling appear during the pronator teres test?  (_______________________________________________________________) |
| **Hawkins kennedy test** | 4. What is the purpose of the Hawkins Kennedy test?  (____________________________________________________________)  5. What is the starting position of the shoulder joint when performing the Hawkins Kennedy test?  (____________________________________________________________)  6. What joint position should the shoulder joint be in when performing the Hawkins Kennedy test?  (____________________________________________________________) |
| **Yergason's test** | 7. When performing Yergason's test, what are the starting positions of the shoulder joint and elbow joint?  (____________________________________________________________)  8. Which of the following is not a disease that can be diagnosed using Yergerson's test? ( )  ① torn transverse humeral ligament (tendon slipping out of groove)  ② humeral glenoid labrum pathology (pain in shoulder)  ③ Strain or tendinitis of the shoulder joint (pain over bicipital groove)  ④ torn rotator cuff muscles  9. What joint position should be taken when performing Yergason's test?  (____________________________________________________________) |
| **Neer's test** | 10. When performing Neer's test, the patient is asked to fully flex the shoulder joint. Please answer whether this is done passively or actively. (_____________________________________________________)  11. What disease can be diagnosed by the Neer's test performed with the palm facing upward?  (____________________________________________________________)  12. What disease can be diagnosed by Neer's test performed with the palm facing down?  (____________________________________________________________) |
